# Flavonoids in Inflammatory Bowel Disease: A Review

**DOI:** 10.3390/nu8040211

**Published:** 2016-04-09

**Authors:** Teresa Vezza, Alba Rodríguez-Nogales, Francesca Algieri, Maria Pilar Utrilla, Maria Elena Rodriguez-Cabezas, Julio Galvez

**Affiliations:** CIBER-EHD, Department of Pharmacology, ibs.GRANADA, Centre for Biomedical Research (CIBM), University of Granada, Avenida del Conocimiento s/n 18016-Armilla, Granada, Spain; teresavezza@hotmail.it (T.V.); albarn@correo.ugr.es (A.R.-N.); fra.algieri@hotmail.it (F.A.); utrillam@ugr.es (M.P.U.); merodri@ugr.es (M.E.R.-C.)

**Keywords:** inflammatory bowel disease, flavonoids, oxidative stress, eicosanoids, barrier function, immunomodulatory properties

## Abstract

Inflammatory bowel disease (IBD) is characterized by chronic inflammation of the intestine that compromises the patients’ life quality and requires sustained pharmacological and surgical treatments. Since their etiology is not completely understood, non-fully-efficient drugs have been developed and those that have shown effectiveness are not devoid of quite important adverse effects that impair their long-term use. In this regard, a growing body of evidence confirms the health benefits of flavonoids. Flavonoids are compounds with low molecular weight that are widely distributed throughout the vegetable kingdom, including in edible plants. They may be of great utility in conditions of acute or chronic intestinal inflammation through different mechanisms including protection against oxidative stress, and preservation of epithelial barrier function and immunomodulatory properties in the gut. In this review we have revised the main flavonoid classes that have been assessed in different experimental models of colitis as well as the proposed mechanisms that support their beneficial effects.

## 1. Introduction

Inflammatory bowel disease (IBD) mainly comprises two major conditions, Crohn’s disease (CD) and ulcerative colitis (UC), characterized by chronic gastrointestinal inflammation with alternating periods of relapses and remissions. Both forms of IBD are featured by exacerbated uncontrolled intestinal inflammation that leads to poor quality of life and requires prolonged medical and/or surgical interventions. Histologically, the examination of intestinal tissue from patients with active disease shows inflammatory cell infiltration corresponding with dramatic tissue injury including edema, loss of goblet cells, fibrosis, erosions and ulcers. In CD patients, the inflammation can intermittently affect any part of the gastrointestinal tract, from the mouth to the anus, but it is usually, although not always, localized in the distal small bowel and/or colon. The inflamed bowel obtained from these patients with active CD reveals transmural inflammation characterized by the presence of large numbers of acute and chronic inflammatory cells within the mucosa, submucosa and muscularis propria [1]. However, UC patients exhibit a non-transmural inflammation, which is restricted, exclusively, to the large bowel and rectum [2]. Typically, the inflammatory changes are limited to the mucosa and submucosa with cryptitis and crypt abscesses, and the inflammatory cell composition is similar to CD. The symptoms associated with both intestinal conditions are largely dependent on disease location and can include diarrhea, abdominal pain, fever, clinical signs of bowel obstruction, as well as passage of blood or mucus or both [1].

**Figure 1:**
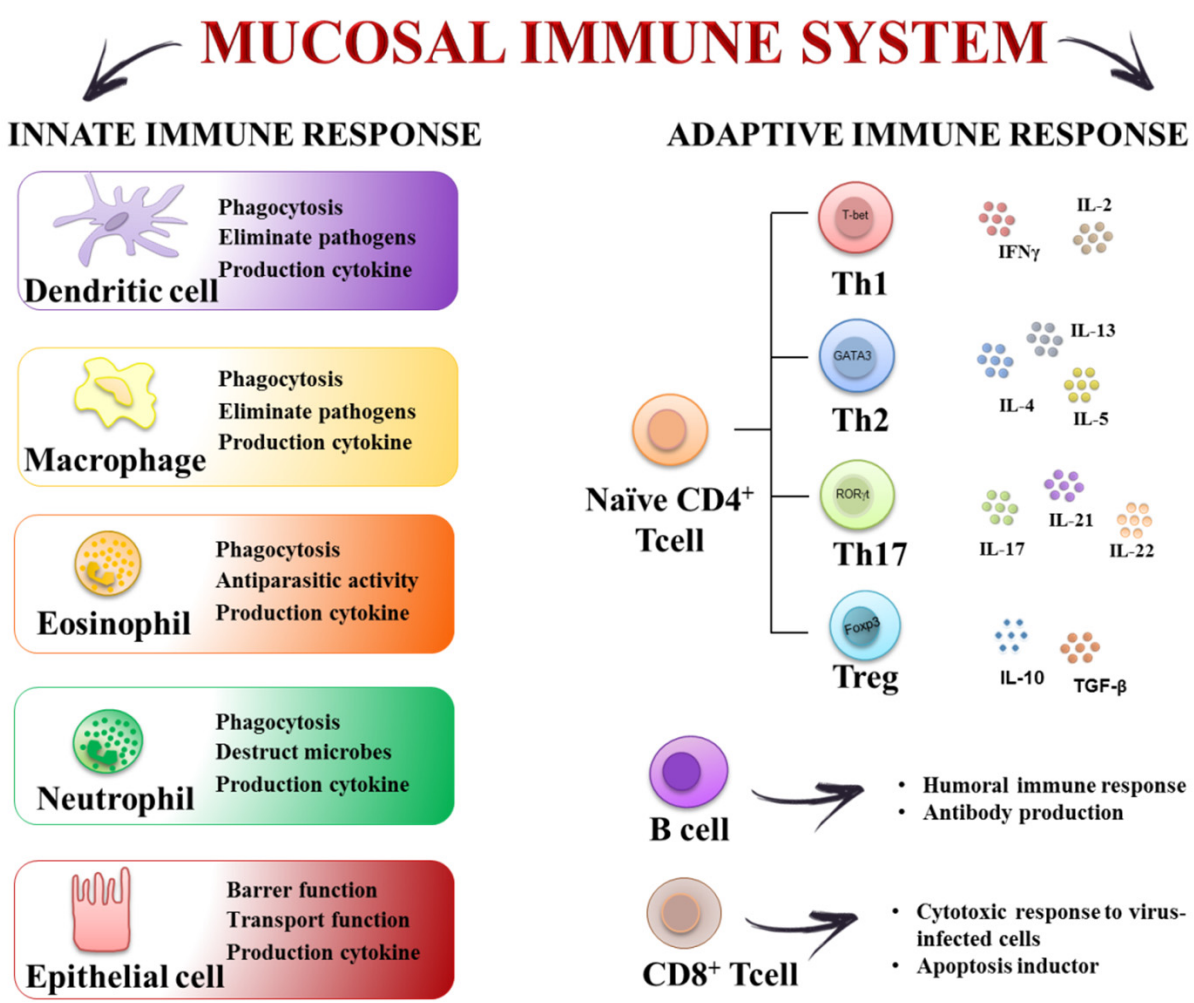
The mucosal immune system constitutes a key element in preventing penetration of microorganisms. It consists of innate and adaptive immune responses. The innate immune response is the first line of defense against infection and includes complement proteins, granulocytes (basophils, eosinophils and neutrophils), mast cells, macrophages, dendritic cells and natural killer cells. The adaptive immune response develops more slowly, but it is manifested as increased antigenic specificity and memory. It consists of antibodies, B cells, and CD4^+^ and CD8^+^ T lymphocytes. Disruption of the innate and acquired gut immune systems may cause the development of chronic intestinal diseases.

At present, the etiology of IBD is not fully understood, and many theories have been proposed to explain IBD pathogenesis, ranging from infectious to psychosomatic, social, metabolic, vascular, genetic, allergic, autoimmune and immune-mediated mechanisms [3,4,5]. Currently, there is a general agreement that IBD occurs in genetically predisposed subjects who exhibit a dysfunctional intestinal epithelium barrier with increased tight junction permeability. In these conditions, these patients develop an exaggerated immune response in the gut towards the intestinal microbiota, which is not conveniently controlled and leads to chronic intestinal inflammation [3]. In fact, various components of the mucosal immune system in the gut have been implicated in the pathogenesis of IBD. They include elements of the innate immune system such as intestinal epithelial cells, macrophages/monocytes, neutrophils, dendritic cells (DCs), as well as constituents of the adaptive immune system such as T-cells and B-cells as well as their secreted mediators (cytokines and chemokines) (Figure 1). It has been proposed that an initial defect in sampling gut luminal antigens, or a mucosal susceptibility, leads to the activation of the innate immune response, most probably associated with an enhanced Toll-like Receptor (TLR) activity. Then, antigen-presenting cells (APCs) can mediate the differentiation of naïve T-cells into effector T helper (Th) cells, including Th1, Th2, and Th17 cell types, and macrophage proliferation, thus impairing the immune tolerance to commensal bacteria in the intestine [6]. In consequence, there is an abnormal synthesis and release of different pro-inflammatory mediators, including eicosanoids, platelet-activating factor, cytokines and reactive oxygen and nitrogen metabolites, which lead to the mucosal damage and the generation of a vicious circle that sustains the inflammatory response that characterizes human IBD [7,8,9].

Nowadays, and since the precise etiology of IBD is unknown, there is no specific causal treatment for these intestinal conditions. For this reason, the main goals of IBD therapy are, firstly, to induce the remission of the symptoms during the acute flare, and secondly, to preserve the remission by controlling the chronic inflammation, thus preventing the reactivation of the intestinal inflammatory process. With these aims, one of the main strategies to effectively counteract the exacerbated immune response is to interfere with multiple stages of the inflammatory cascade, mainly by using aminosalicylates (sulfasalazine or mesalazine), immunosuppressants (glucocorticoids, azathioprine, methotrexate, and cyclosporine A), and biologicals (infliximab or adalimumab) [10]. Unfortunately, these treatments are not devoid of potentially serious side effects, thus limiting their chronic use [11]. In consequence, there is a clear demand for safe and effective therapeutic strategies for human IBD. This could be the case for flavonoids, natural phenolic products that are found in edible fruit and vegetables and present several biological activities, mainly related to their antioxidant properties and ability to inhibit enzymes, which justify their reported capacity to downregulate the immune response [12]. Therefore, they could be taken into consideration as potential drugs for the pharmacological treatment of IBD. The aim of this review is to provide scientific arguments that would support the use of flavonoids in the treatment of human IBD, based on different studies that have shown the efficacy of these compounds both in clinical trials and in an experimental model of rodent colitis. Moreover, we will analyze the mechanisms that may be involved in their beneficial effects in these intestinal conditions. With this purpose we will focus on the most relevant groups of flavonoids with demonstrated intestinal anti-inflammatory properties: anthocyanidins, catechins, chalcones, flavanones, flavones, flavonols, isoflavones.

## 2. The Inflammatory Response in the Gut: Cellular and Molecular Mechanisms

The gastrointestinal tract is key for the maintenance of immune homoeostasis, and any dysregulation could result in different pathologies, including IBD. The intestinal innate immune system is the first line of defense, thus providing an immediate protective response against infections, and it also helps to initiate the adaptive immune response. The innate immune system comprises the epithelial cell barrier, macrophages, monocytes, neutrophils, DCs, natural killer (NK) cells, eosinophils and basophils. These cells act together to confer tolerance or to initiate inflammation by secreting cytokines, chemokines and antimicrobial agents. Besides, the surface of the intestine is protected by a layer of mucus that is generated by the goblet cells of the epithelium.

It has been proposed that one of the early steps that occur in IBD is related to the disruption of the epithelial-cell barrier that may result in inflammation and dysregulation of the mucosal homeostasis. In this setting, some of the cells involved in innate immunity, such as macrophages and DCs, are able to identify microorganisms’ molecular patterns by using the corresponding pattern recognition receptors (PRR), including TLRs [13]. This promotes the recruitment of monocyte-derived macrophages that generate cytokines and chemokines to attract monocytes and other leukocyte populations in an attempt to contain the inflammation [14]. When innate immunity fails to counteract the pathogen aggression, the adaptive immune response is triggered. In this process, DCs get into the mesenteric lymph nodes, and then they present the antigen to naive T cells and, depending on the factors released by DCs, induce the T cell differentiation [15]. T cells are the key players of the adaptive immune response, and in collaboration with other cells and molecules from the innate immune system, are able to trigger an effective response to remove the invading pathogens. Depending on the effector cytokines produced by APCs, naive T cells have the potential to differentiate into different T helper (Th) subtypes. IL-12 generates Th1 cells, IL-4 promotes Th2 differentiation, IL-10 and TGF-β induce regulatory T cells (iTreg), whereas IL-6, IL-1β and TGF-β promote Th17 cells [16,17,18,19]. It has been reported that these Th subtypes exhibit specific functions; whereas Th1 cells are crucial for removing intracellular pathogens, Th2 cells mediate allergic reactions and protect against parasites, and Th17 cells contribute to eliminating extracellular bacteria and fungi [20,21]. In addition to Th cells, neutrophils are also able to infiltrate the inflamed mucosa. These activated leukocytes generate several pro-inflammatory cytokines, but they also play a key role in inducing oxidative reactions, thus modifying the redox balance in the gut mucosa, which can collaborate to maintain the inflammatory status through the upregulation of redox-sensitive signaling pathways and transcription factors [22]. Furthermore, all the inflammatory molecules involved in these intestinal conditions produce additional oxidation products, thus resulting in a self-sustaining and auto-amplifying vicious circle, which ultimately worsens the already-compromised gut barrier integrity. Accordingly, and similarly to other inflammatory disorders, the complex immune cell response in IBD implies the participation of an extensive variety of inflammatory mediators, including cytokines, chemokines, leukotrienes and prostaglandins. All of them contribute actively to the inflammatory process at different stages: initiation, progression and resolution, if it happens.

It is well reported that there is increased expression of chemokines in the active phases of IBD, including IL-8, monocyte chemoattractant protein (MCP)-1 and MCP-3, and macrophage inflammatory proteins (MIP), which are responsible for the recruitment of different leukocyte effector populations, by controlling their adhesion and migration across the endothelium in sites of inflammation. Moreover, these mediators are also able to trigger other inflammatory processes such as leukocyte activation, granule exocytosis, activation of metalloproteinases for matrix degradation and upregulation of the oxidative burst, which are actively involved in the tissue injury that occurs in intestinal inflammation [23]. Likewise, there is upregulated expression and release of different adhesion molecules in IBD, such as the intercellular adhesion molecule (ICAM)-1, the lymphocyte function-associated antigen (LFA)-1, the macrophage 1 antigen (Mac-1), the vascular cell adhesion molecule (VCAM)-1, the very late antigen (VLA)-4 and P- and E-selectins, which also participate in the recruitment of leukocytes to the inflamed tissue [24].

When considering the cytokines, their roles in IBD are very diverse and complex, and they have a key role in controlling T-cell differentiation and regulation. Thus, they are considered as crucial targets to control the inflammatory response. IL-12, IL-18 and IL-23 are responsible for Th1 differentiation and chronic activation, whereas other cytokines, such as TNFα, IL-1β and IL-6, enhance the inflammatory response by promoting the recruitment and activation of other inflammatory elements, thus increasing the production and release of inflammatory mediators [25]. Additionally, the activation of PRRs induces the expression of IL-23, which enhances both Th1 and Th17 responses, this being associated with an increased production of other cytokines such as IFNγ, IL-17 and IL-22 in chronic intestinal inflammation [26,27]. The IL­17 cytokine family comprises a group of at least six members, IL-17A, IL­17B, IL­17C, IL­17D, IL­17E (or IL­25) and IL­17F, which have been reported to display potent pro-inflammatory responses both *in vitro* and *in vivo* [28]. In fact, IL-17 increases the expression of pro-inflammatory cytokines, including IL­6 and TNFα, chemokines, such as the keratinocyte chemoattractant (KC), MCP-1 and MIP-2, and matrix metalloproteases, all of them involved in tissue infiltration and tissue damage [29], and thus revealing a crucial function in the pathogenesis of human IBD [30]. Finally, it is important to note that an altered production of anti-inflammatory cytokines, such as IL-10 and TGFβ, can also contribute to the pathogenesis of IBD, given the key role attributed to these cytokines as regulators of intestinal immune homeostasis and the inflammatory responses [31,32].

## 3. Intestinal Anti-Inflammatory Effects of Flavonoids: *In Vivo* and *in Vitro* Studies

**Figure 2:**
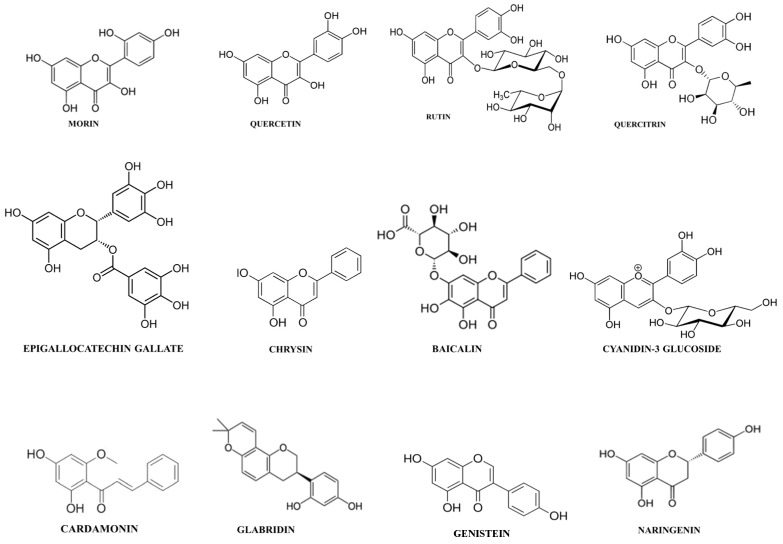
Chemical structures of the main flavonoids with intestinal anti-inflammatory properties.

The biological activities ascribed to flavonoids, including antioxidant properties and the inhibition of enzymatic activities, can justify the fact that many flavonoids have been described to suppress inflammation, both *in vivo* and *in vitro*, thus reducing the severity of different inflammatory diseases, including IBD [33,34]. Galsanov *et al.* (1976) reported for the first time the potential beneficial effect of flavonoids in intestinal inflammation. In that study, the authors described the anti-inflammatory activity of quercitrin, when administered at doses of 25 and 100 mg/kg, in a rat model of allergic intestinal inflammation. Since then, many studies describing the impact of flavonoids in several experimental models of colitis in rodents have been published. Among these, chemically induced models (acetic acid, trinitrobenzenesulfonic acid (TNBS) or dextran sulphate sodium (DSS)), genetically engineered mice (HLA-B27 rats or IL-10 knock-out (KO) mice), and a T cell–transfer model have been used broadly and have been shown to share some similarities with human IBD [35]. These studies have revealed the intestinal anti-inflammatory activity of different flavonoids, including both glycosides and aglycones, and those belonging to the different chemical classes, such as flavonols (quercetin, quercitrin, rutin), flavanones (naringenin), flavones (baicalin, chrysin), catechins (epigallocatechin-3-gallate (EGCG)), isoflavones (genistein, daidzein, glabridin), anthocyanidins (cyanidin-3-glucoside (C3G)), and chalcones (cardamonin) (Table 1) (Figure 2). These beneficial effects were evidenced at acute and semi-chronic stages of intestinal inflammation, following either a preventative dosing protocol, *i.e.*, when administered before colitis induction or a curative administration of the test compounds once the colonic damage had been developed. At present, it is difficult to establish a structure-activity relationship, since the number of flavonoids tested until now is low. However, of all the flavonoids tested, quercitrin has been found to be the most potent, showing preventative or curative properties at doses of 1 and 5 mg/kg [36]. The range of active doses for the other flavonoids is broad, ranging from 10 to 25 mg/kg when the glycosides are considered, and between 10 and 200 mg/kg in the case of aglycones.

At present, it is not completely clear whether these beneficial effects are due to a local action of the flavonoid on the inflamed colonic tissue or whether they are derived from a systemic effect after their absorption in the small intestine and subsequent metabolism, or both. For instance, regarding the flavonol quercetin, the oral administration of its aglycone, at doses of 9 mg/kg [38], is devoid of any beneficial effect, whereas its derived glycoside, quercitrin, shows intestinal anti-inflammatory effects in different models of experimental colitis at doses under 5 mg/kg [36,39]. The aglycones are absorbed in the small intestine while most glycosides reach the colon, where they are cleaved by the microbiota and the moiety of the aglycone is released. This could explain why the glycoside derivatives could be more potent on colonic inflammation when they are administered orally.

The flavonoid treatments have shown beneficial effects that were evidenced macroscopically by the amelioration of colonic damage. In the DSS model, characterized by intense colonic damage associated with inflammatory cell infiltration and epithelial crypt loss, which results in acute clinical symptoms including body weight loss, bloody stools and diarrhea, the treatment with some flavonoids such as cardamonin, chrysin, naringenin, EGCG, glabridin, rutin and quercitrin significantly reduced the Disease Activity Index (DAI), which is used to monitor the severity of the inflammatory process, as well as the colon shortening. Therefore, DSS-induced weight loss and histological damage were significantly ameliorated by flavonoid treatment [40,50,55,56,57,58,59,60]. In line with this finding, it has been shown that quercitrin and rutin administration was able to decrease colonic damage, reducing the length of the injury and attenuating the diarrhea symptoms in the TNBS model [61,62]. Moreover, rutin also ameliorated histologic injury in the acetic acid model of experimental colitis [63].

The involvement of different mechanisms in the intestinal anti-inflammatory activity of the flavonoids has been proposed, including antioxidant properties through interference with reactive oxygen (ROS) and nitrogen (RNS) species, inhibition of eicosanoid synthesis, immunomodulatory activity, preservation of the epithelial barrier function and, finally, an interference with the gut microbiota.

### 3.1. Antioxidant Properties of Flavonoids

Several studies have proposed that both ROS and RNS also play a key role in the etiology of IBD [64]. In fact, human IBD has been associated with an intense oxidative stress, excessive generation of ROS and RNS in the intestinal tissue which induces lipid peroxidation, protein modifications, DNA damage, and apoptosis, together with impairment of the enzymatic and non-enzymatic antioxidant mechanisms, including superoxide dismutase (SOD), and reduced glutathione (GSH) and catalase (CAT), which results in the colonic damage associated with intestinal inflammation [22,65]. Different sources of free radicals have been proposed to contribute to the oxidative burst that takes place in IBD, with neutrophils among the cells most involved in these processes [54]. The infiltration of polymorphonuclear neutrophils and mononuclear cells into the affected part of the intestine is considered one of the main pathological features of human IBD [66]. As a consequence of the activation of the nicotinamide adenine dinucleotide phosphate (NADPH) oxidase system in these cells, and the subsequent myeloperoxidase activity (MPO), massive quantities of superoxide and hypochlorous acid are generated and cause direct cytotoxicity in the intestinal tissue. This, in turn, facilitates the additional release of different pro-inflammatory mediators [48]. In fact, most flavonoids assayed in experimental colitis models exhibited a significant reduction of colonic myeloperoxidase. This enzyme is predominantly found in the azurophilic granules of the neutrophils and is considered to be a sensitive marker of leukocyte infiltration [67]. As expected, MPO activity is increased in different experimental models of colitis induced by TNBS, DSS and T cell transfer. The increased MPO levels were significantly reduced after the administration of genistein and quercitrin in the TNBS model [61,68]. A similar effect was induced by quercitrin as well as with cardamonin, chrysin, EGCG, naringenin and rutin in the DSS model [40,50,55,56,57,60,69]. Finally, it is important to remark that rutin administration was also able to reduce leukocyte infiltration in the T cell transfer model [70].

Most of the flavonoids assayed were able to ameliorate the oxidative stress that takes place in the experimental models of colitis as evidenced by a reduced colonic lipid peroxidation, together with an improvement in different antioxidant markers, including sulfhydryl-derived compounds, or an enhancement of different enzyme activities with antioxidant properties [69]. Specifically, several studies have suggested that both EGCG and quercitrin administration on the DSS-induced colitis model were able to increase the colonic GSH production, and naringenin and EGCG reduced the tissue malondialdehyde (MDA) levels, indicating both a reduction of lipid peroxidation and an increase of antioxidant enzymes such as SOD and GPO [55,56,60,71]. Similarly, quercitrin and rutin treatment have shown to significantly increase GSH levels, thus ameliorating the colonic damage in the TNBS model [61].

**Figure 3:**
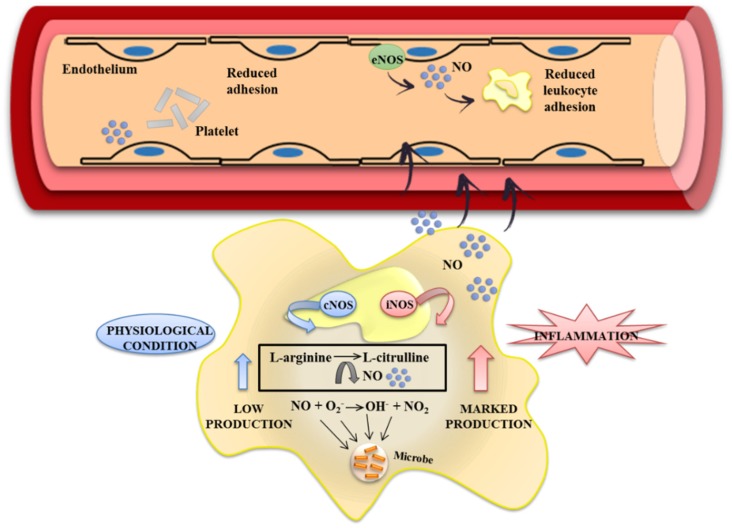
Nitrite oxide (NO) is a free radical molecule generated from l-arginine oxidation, and is catalyzed by the enzyme nitric oxide synthase (NOS). Different functional forms of NOS can be recognized: constitutive and inducible forms. NO synthesis by the constitutive isoform, endothelial NOS (eNOS), generates low levels of NO under normal physiological conditions which regulates the colon blood flow, bowel motility and produces reactive oxygen species (ROS) for fighting pathogens. The inducible isoform, iNOS, is expressed in cells involved in the inflammatory response and, upon different stimuli, generates high levels of NO that may be toxic to the healthy tissue, contributing to damage and upregulation of the inflammatory response. Several studies clearly demonstrated that certain flavonoids inhibit NO production in activated cells and in induced experimental colitis. Their inhibitory activity might be due to reduction of iNOS enzyme expression.

Special attention can be paid to the RNS, which can be produced and released by immune cells and also play an important role in the pathophysiology of IBD. Nitric oxide (NO) is a pleiotropic free radical messenger molecule produced from L-arginine by the nitric oxide synthase (NOS) enzyme. Under physiological conditions, low levels of NO are produced by the isoform of constitutive nitric oxide synthase (cNOS), which has a direct protective effect throughout the initial phases of the intestinal inflammatory process. Nevertheless, in chronic inflammation, NO synthesis is upregulated, mainly as a consequence of the increased expression of the inducible isoform of nitric oxide synthase (iNOS), which is induced, mainly in macrophages, by bacterial products and pro-inflammatory cytokines [47,72]. The overproduction of NO contributes to colonic damage due to its interaction with the superoxide anions, thus generating peroxynitrites, which reinforce oxidative stress and tissue damage [41] (Figure 3). Numerous studies have described an effect of flavonoids on the metabolism of NO, which may preserve the beneficial functions of NO through the direct capture of super oxide anions [73]. Similarly, it has been reported that flavonoids are capable of inhibiting the expression of iNOS [60] as well as acting as powerful captors of peroxinitrite radicals [74]. Moreover, DSS administration is associated with a significant increase of iNOS. In this regard, it has been observed that some flavonoids such as glabridin, cardamonin, naringenin and quercitrin improve the inflammatory process, reducing the expression of iNOS and, as a consequence, the NO production [40,58,59,60]. These results have been confirmed in *in vitro* studies with different cell lines. EGCG, naringenin, daidzenin, kaempferol, quercetin and cardamonin inhibit iNOS protein and mRNA expression and also NO production in lipopolysaccharide (LPS)-activated macrophages, such as bone marrow–derived macrophages (BMDM), or murine macrophages J774 and mouse leukemic monocyte macrophage (RAW 264.7) cell lines [39,40,71,75]. Flavonoids are capable, therefore, of preventing the detrimental effects generated by NO in intestinal inflammation.

### 3.2. Effects of Flavonoids on Eicosanoid Metabolism and Function

Eicosanoids derived from arachidonic acid metabolism, including products from cyclooxygenase (COX) (prostaglandins) and lipoxygenase (LOX) (leukotrienes), seem to also play a critical role in intestinal inflammation. In fact, it has been demonstrated that increased levels of eicosanoids are found in the inflamed tissue areas in comparison with normal mucosa in human IBD [76]. Actually, the upregulation of the enzymes involved in the eicosanoid metabolism has also been associated with the pathophysiology of other inflammatory disorders [77].

**Figure 4:**
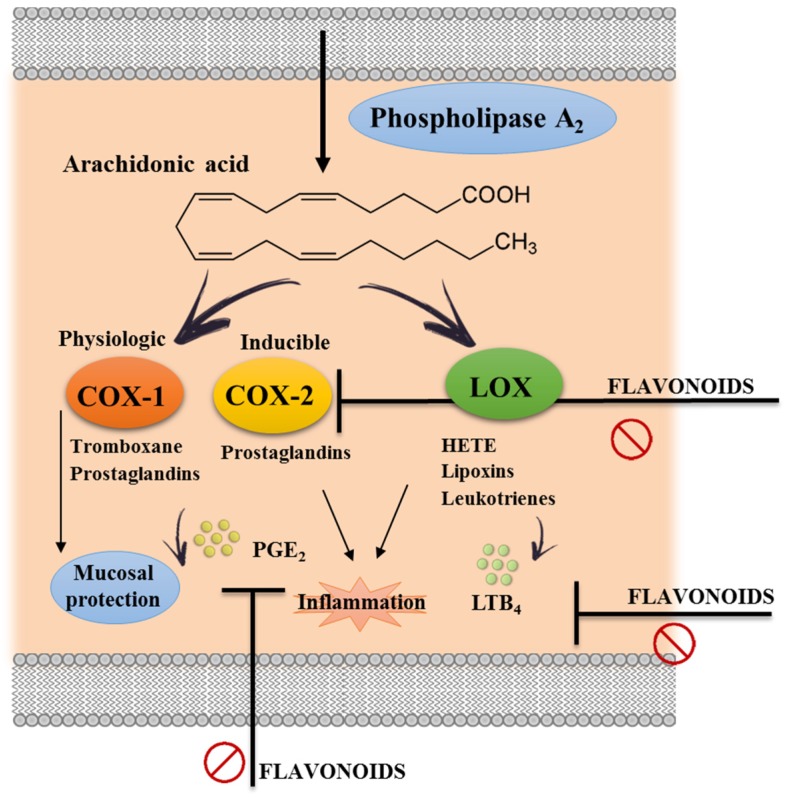
Eicosanoid synthesis pathways: Arachidonic acid is a polyunsaturated fatty acid that is released from the cellular membranes by cytoplasmatic phospholipase A_2_ (PLA_2_). Free arachidonic acid can be metabolized to eicosanoids through two major pathways: the cyclooxygenase (COX) and the lipooxygenase (LOX). The COX-1 (constitutive form) pathway results in the synthesis of prostaglandins and thromboxanes, which are important for physiological functions. The COX-2 (inducible form) pathway plays a crucial rule in the production and release of inflammatory prostaglandins. Similarly, the LOX pathway leads to the synthesis of leukotrienes and hydroxyeicosatetraenoic acid (HETE) that contribute to the inflammatory process. Different studies have associated the flavonoid anti-inflammatory effect with a suppression of these pathways.

There are two isoforms of COX: constitutive COX-1 and inducible COX-2. COX-1 has been considered crucial for mucosal integrity since it produces cytoprotective and anti-inflammatory prostaglandins such as PGE_2_ [37]. On the contrary, the expression of COX-2 can be induced by a variety of stimuli related to the inflammatory response. This isoform is responsible for an increased production of prostaglandins involved in IBD [78,79] (Figure 4). In consequence, and theoretically, the inhibition of COX-2 expression and/or activity would be also beneficial in the management of intestinal inflammation. Different studies have reported that the intestinal anti-inflammatory activity of flavonoids, such as rutin and EGCG, was associated with the inhibition of the colonic expression of COX-2 [58,71].

Interestingly, it was reported that C3G has inhibitory effects on the production of several mediators during inflammation in the colonic carcinoma cell line HT29, in comparison with 5-aminosalicylic acid (5-ASA), a well-established anti-inflammatory drug used in IBD. In this regard, treatment with 25 mM C3G, 500 mM 5-ASA or both, for one hour, before cytokine (IL-1α, TNFα and IFNα) stimulation, significantly reduced PG_2_ production. C3G produced the strongest inhibition (65%) while 5-ASA produced a significantly lower inhibition (50%). Additionally, C3G downregulated COX-2 expression more efficiently than 5-ASA, and the combination of C3G and 5-ASA afforded a much better protection than that of the individual compounds [80].

On the other hand, the increased generation of leukotrienes, mainly LTB_4_, has also been reported to occur in IBD [81]. In this regard, it has been proposed that leukotrienes mediate the intestinal inflammatory response, especially through their chemotactic effects, thus inducing the accumulation of inflammatory cells in the inflamed area of the gut. In consequence, the inhibition of lipoxygenase activity and the subsequent reduction of LTB_4_ production, or the blockade of its receptor, could be proposed to exert beneficial effects in experimental colitis [46,82]. However, although different flavonoids with beneficial effects in experimental colitis were able to reduce colonic LTB_4_ production, no direct relationship between the reduced levels of this eicosanoid in the colonic tissue and the anti-inflammatory effect can be established [36,53].

### 3.3. Immunomodulatory Properties of Flavonoids

As mentioned previously, most of the studies performed in experimental models of colitis have proposed that an imbalance of the immune system plays a key role in IBD pathogenesis. The altered immune response is associated with an increased release of pro-inflammatory cytokines, including IFN**γ**, TNFα, IL-6, IL-1β, GM-CSF and IL-17A, chemokines, such as IL-8, MIP-2 and MCP-1, and adhesion molecules, such as ICAM-1. The ability of flavonoids to regulate the altered immune response that occurs in intestinal inflammation has been reported in different *in vivo* studies. For instance, the administration of flavonoids, such as EGCG, cardamonin, chrysin, glabridin, quercitrin, naringenin or rutin, in the DSS model remarkably decreased the increased levels of the different cytokines evaluated in the inflamed colon [40,44,55,56,57,58,59,60].

These immunomodulatory properties exerted by the flavonoids have also been confirmed when *in vitro* experiments were performed in different cell types involved in the immune response: epithelial cells, monocytes/macrophages, T cells, and dendritic cells. For instance, the incubation of LPS-activated macrophages, RAW 264.7 and BMDM cells with quercetin or baicalin resulted in reduced levels of IL-1β and TNFα when compared with stimulated cells without flavonoid treatment [39,83]. Similarly, rutin was able to significantly reduce increased IL-1β levels produced by DSS-stimulated pMφ cells, obtained from mouse peritoneal exudate [58,84]. In addition, quercetin exerts anti-proliferative effects by reducing IFNγ and TNFα production in concavalin A-stimulated purified T lymphocytes isolated from rat splenocytes [70]. Moreover, the incubation of flavonoids, such as C3G, genistein, EGCG or chrysin, in cytokine-stimulated epithelial cells, Caco-2 and HT-29 cells significantly reduces IL-8 secretion in the cell culture [80,85]. Finally, it has been shown that treatment of THP-1 cells, a human monocytic cell line, with EGCG decreased MCP-1 and CCR2 gene expression, together with MCP-1 secretion and CCR2 expression, at the cell surface, and induced the inhibition of beta1 integrin activation [45].

Considering the role of immune cells in the development of IBD, T cells are major players [86]. Likewise, DSS-induced experimental colitis has been associated with an increased percentage of Th1 and Th17 cells in the mesenteric lymph nodes, which correlates to the overexpression of pro-inflammatory cytokines such as IFNγ, IL-17A and IL-17F. In this regard, it has been reported that the beneficial effects observed with the flavonoid derivative icariin in DSS-induced colitis in mice were related to a downregulation of the proportion of both Th1 and Th17 cells, and thus a reduction in the cytokine release by these cell subtypes in the colonic tissue [87]. Different *in vitro* studies have also confirmed the ability of flavonoids to suppress T cell proliferation and activation [87,88,89]. Moreover, baicalin was able to reduce the expression of RORC, Foxp3 and T-bet, transcription factors associated with Th17, Treg and Th1 cells, respectively, which have been reported to be upregulated in UC patients [90]. Similarly, macrophages have been considered to be the main source of different pro-inflammatory mediators in IBD, including TNFα, IL-1β and NO, thus actively contributing to the pathology of these intestinal conditions [91,92]. Besides, several *in vitro* studies have shown the capacity of flavonoids to inhibit NO and cytokine production in different macrophage cell lines, including RAW 264.7 and J774.1, as well as in bone marrow–derived macrophages (BMDM) [33,93]. *In vivo* experiments have also revealed that the beneficial intestinal anti-inflammatory effects of flavonoids, such as quercitrin, were associated with a decreased number of infiltrated macrophages in the inflamed colonic tissue induced by DSS in rats [60]. Finally, and as mentioned above, neutrophil infiltration can be considered one of the main pathological features of human IBD [66], and most active flavonoids assayed in experimental colitis models significantly reduced neutrophil infiltration into the damaged colonic tissue, as evidenced by a significant reduction of colonic myeloperoxidase [40,50,55,56,57,60,61,68,69,70], thus contributing to the amelioration of the intestinal inflammation.

**Figure 5:**
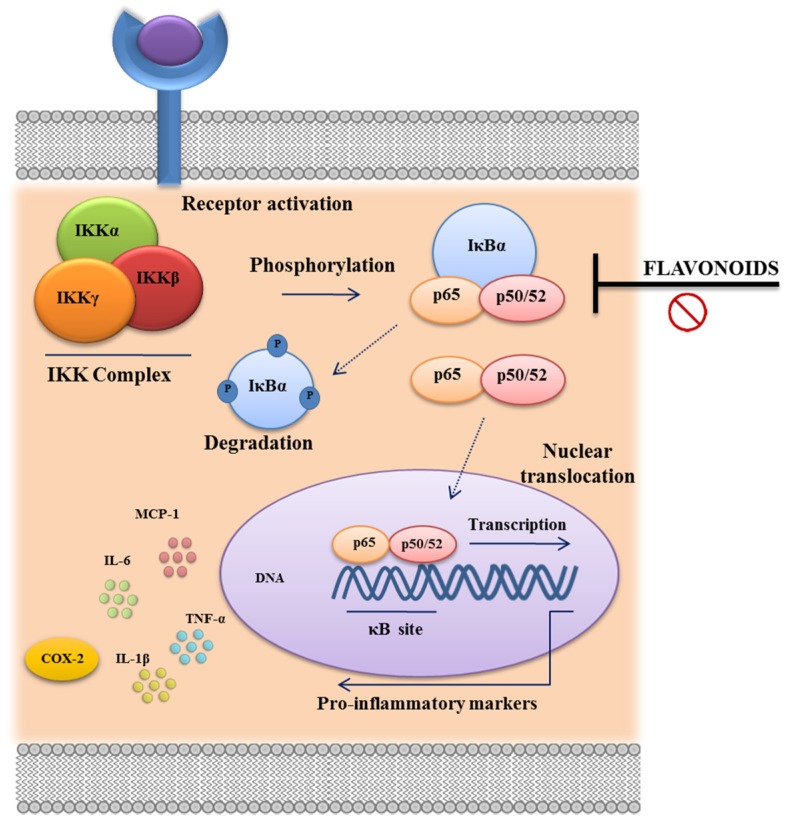
NF-kB signal transduction pathway. NF-κB protein complex (p65–p50) is bound and inhibited by IκB proteins. Pro-inflammatory cytokines, LPS, growth factors, and antigen receptors activate an IKK complex (IKKβ, IKKα, and IKKγ), which phosphorylates IκB proteins. Phosphorylation of IκB leads to its ubiquitination and proteasomal degradation, releasing NF-κB. Active NF-κB proteins are further activated by post-translational modifications (phosphorylation, acetylation, glycosylation) and translocate to the nucleus where they induce target gene expression, influencing a broad range of biological processes including innate and adaptive immunity, inflammation, stress responses, B cell development, and lymphoid organogenesis. Anti-inflammatory effects of several flavonoids have been related to the suppression of the NF-κB signal transduction pathway.

Several studies have focused on the potential mechanisms responsible for the modulation of cytokine production; some of the mechanisms proposed are related to the inhibition of nuclear factor-κB (NF-κB), mitogen-activated protein kinase (MAPK) and STAT activation [94,95,96,97]. NF-κB is ubiquitously expressed, being found in its inactive form in the cytoplasm, which is bound to its high-affinity inhibitor IκB. In the presence of an activating stimulus, including oxidative stress, a large signaling cascade is initiated, resulting in the activation of IKK-α and IKK-β, two kinases that phosphorylate IκB. Phosphorylation of IκB results in its dissociation, and NF-κB becomes free to translocate to the nucleus, where it binds to κB regulatory elements, activating gene expression [98] (Figure 5). It has been clearly demonstrated that cardamonin and quercitrin exerted potent anti-inflammatory properties by reducing NF-κB activity in DSS-induced experimental colitis, whereas rutin reduced IκBα phosphorylation in a T cell transfer model [51,60,70]. Baicalin has also shown the ability to block this pathway in the TNBS model of rat colitis [83]. In this regard, different studies have proposed that the ability of some flavonoids to downregulate the altered immune response that occurs in intestinal inflammation may be achieved through the inhibition of the TLR4/NF-κB signaling pathway, as it has been demonstrated *in vitro*, when activated mouse macrophage J774 and RAW264.7 cells or human colonic HT-29 cells were exposed to naringenin, kaempferol, quercetin, daidzein and cardamonin [71,75,99,100]. Similarly, it has been reported that EGCG reduced LPS-induced TNFα production in macrophages (RAW264.7 cell line and peritoneal macrophages) by blocking NF-κB activation. In the case of baicalin and cardamonin, this effect was associated with the inhibition of NF-κB p65 subunit phosphorylation [40,83,101,102], whereas quercetin was able to reduce the IκBα phosphorylation in LPS-activated BMDM [39]. The flavonoids’ ability to interfere with NF-κB phosphorylation has been also demonstrated in IEC-6 cells (an intestinal epithelial cell line), and in peripheral blood mononuclear cells (PBMC), Caco-2 and BMDM cells. Specifically, baicalin, chrysin, quercetin, and EGCG administration showed inhibition of the NF-κB pathway through other mechanisms [44,50,85,103,104].

**Figure 6:**
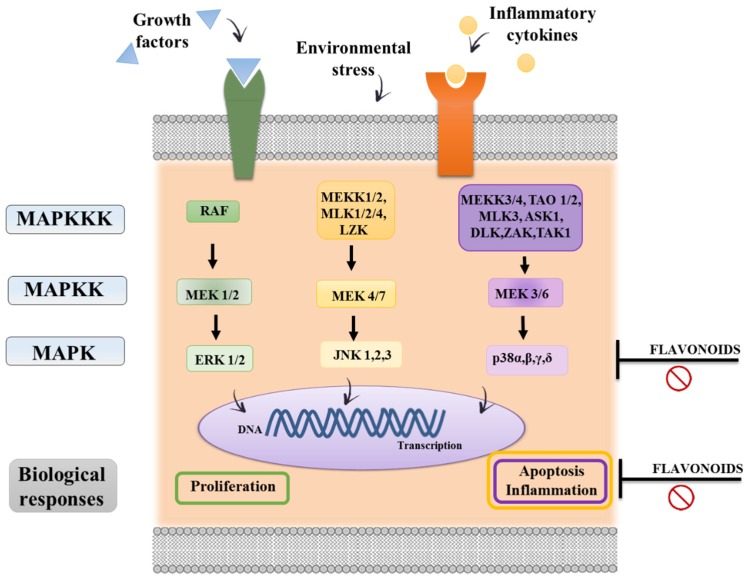
MAP Kinase pathway. The mitogen-activated protein kinase (MAPK) cascades are signal transduction pathways that involve a chain of three kinases activating each other in a series (MAPKKK, MAPKK, and MAPK). The result of phosphorylation of various MAP kinase isoforms is the activation of the three main MAP kinases: ERK (extracellular signal-related kinase), p38 MAPK, and JNK (c-Jun NH2-terminal kinase). Cell division, migration, and survival generally involve ERK signaling. Cellular stress activates the p38 MAPK and JNK pathways. The p38 MAPK pathway mediates transcription and cell motility. JNK signaling regulates apoptosis and inflammation. Flavonoids’ immunomodulatory properties may be related to a direct inhibitory effect on the kinases themselves or by modulation of signal transduction events upstream of the relevant MAPK pathways.

The MAPK signaling pathway also promotes immediate early gene and transcription factor activation of cellular responses such as cytokine production, apoptosis and migration. A general feature of MAPK pathways is the participation of a three-tiered kinase canonical cascade consisting of a MAPK, a MAPK kinase (MAPKK) and a MAPK kinase kinase (MAPKKK) [105,106] (Figure 6). Different *in vitro* studies have associated the flavonoid anti-inflammatory effect with a suppression of this pathway; for instance, EGCG was able to suppress the maturation of murine dendritic cells through the inhibition of extracellular signal-regulated kinase, p38 kinase and c-Jun NH_2_-terminal kinase [107].

**Figure 7:**
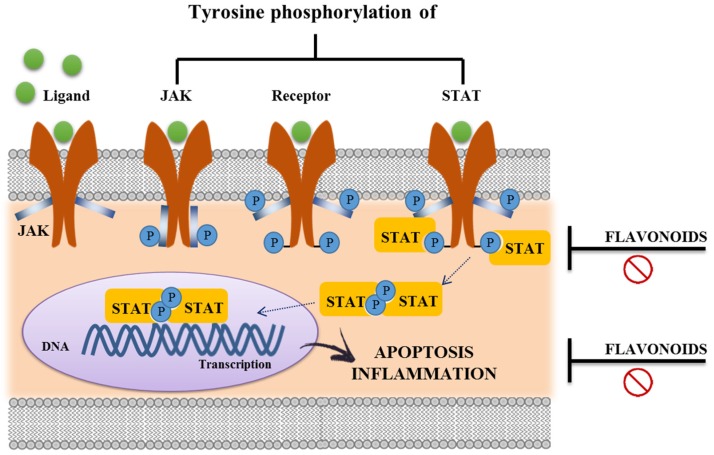
JAK-STAT signaling pathway. Upon the binding ligand, receptor-associated Janus Kinases (JAKs) become activated and mediate phosphorylation of specific receptor tyrosine residues. This leads to the recruitment of specific STATs (Signal Transducers and Activators of Transcription), which are then also tyrosine-phosphorylated. Activated STATs are released from the receptor, they dimerize and translocate to the nucleus to bind target genes associated with proliferation, differentiation and survival of the cells, including immune cells. The inhibition of JAK-STAT signaling pathway by flavonoids treatments leads to an immunomodulatory effect.

Finally, the JAK/STAT pathway transduces signals from a wide range of extracellular cytokine stimuli to the nucleus in order to orchestrate an appropriate cellular response through target gene expression [108,109]. The binding of cytokines to their corresponding transmembrane receptors induces receptor dimerization of its subunits and association with JAK tyrosine kinases. Once activated, STAT proteins dissociate from the receptor, homo- or heterodimerize, and rapidly translocate from the cytoplasm into the nucleus. Thus, the JAK/STAT cascade provides a direct mechanism to translate an extracellular signal into a transcriptional response [110] (Figure 7). Many flavonoids can inhibit both JAK/STAT signaling, kaempferol and EGCG [49,75,111]. Interestingly, Western blotting analysis in HT29 cells suggested that anthocyanin cyaniding-3-glucoside remarkably reduces cytokine-induced levels of activated STAT1 [80], whose expression and activation have been shown to be upregulated in IBD patients [112].

### 3.4. Effects of Flavonoids on Intestinal Barrier Function

The homeostasis in the gastrointestinal tract is functionally maintained by an epithelial barrier, composed by a selective monocelular layer between the outside lumen and host tissues, which controls the equilibrium between tolerance and immunity to microbes and non-self-antigens. Several defects related to intestinal barrier function have been found in IBD patients. Whether mucosal barrier impairment is a consequence of the inflammatory response or a primary defect that prompts mucosal inflammation is still under debate [113]. However, transgenic animal models have clearly demonstrated that a unique defect in the intestinal epithelial barrier is enough to trigger the development of chronic gut inflammation [42]. In addition, several studies suggest that the impairment of the epithelial barrier function can be considered as one of the early events that occur in intestinal inflammation, since it facilitates the entry of antigens from the intestinal lumen to the mucosa that may prompt the uncontrolled and exacerbated immune response [114,115]. For this reason, its recovery may contribute to the beneficial effects produced by flavonoids in experimental colitis models. It has been reported that different flavonoids such as quercitrin [36], rutin [63], hesperidin [116] and morin [53] improve the colonic absorptive function greatly compromised in experimental colitis, leading to fewer diarrhea symptoms, which are frequent in intestinal inflammation. The flavonoid anti-diarrheal effects have also been related to their capacity to inhibit muscle contractility, enhance intestinal motility and reduce fluid intraluminal accumulation in the gut lumen, as evidenced in different experimental studies [117,118,119].

Moreover, Azuma *et al.* (2013) [59] also reported that naringenin treatment in colitic mice resulted in an improvement in the epithelial barrier permeability, through the preservation of the intestinal tight junction barrier function and structure, which have been described to be compromised after DSS administration [120,121]. *In vitro* studies have confirmed the ability of flavonoids, such as naringenin, daidzenin and morin, to enhance epithelial barrier function. In particular, the incubation of these flavanones with human intestinal Caco-2 epithelial cells resulted in an increased transepithelial electrical resistance (TER) across the cell monolayers, which correlates to an improvement of tight junction integrity [122]. This was confirmed by immunoblot analysis and confocal microscopy, which demonstrated that naringenin, daidzenin and morin increase the cytoskeletal expression of the tight junction proteins as well as their assembly, thus reinforcing epithelial integrity in this cell line [122]. In addition to a direct effect on tight protein function, indirect mechanisms can also account for the beneficial effects of flavonoids in preserving the intestinal barrier function. In fact, it has been reported that pro-inflammatory cytokines, such as IFN**γ**, TNFα or IL-6, can disrupt the epithelial barrier function by apoptosis-independent mechanisms [43,123]. In consequence, the inhibitory effect exerted by these compounds on the expression of IFN**γ** and IL-6 can also contribute to the improvement of the intestinal permeability observed in DSS experimental colitis [59].

### 3.5. Interaction of Flavonoids with Gut Microbiota

As commented previously, there is increasing experimental evidence that supports the role of luminal bacteria in the initiation and development of the intestinal inflammatory process, which would be probably related to an imbalance in the intestinal microbiota composition, known as dysbiosis [52,124]. In fact, previous studies have tried to explain the alterations that occur in the gut microbiota or identify the bacterial populations that might be associated with the onset or recurrence of IBD, thus promoting the access of potential pathogens to the lamina propria and triggering the exacerbated immune response [3,125,126,127,128]. Different studies have reported that diets containing bioactive compounds, such as phenolic compounds and tannins, can be considered as possible complementary treatments for IBD due to their antimicrobial and antioxidant capacity [129]. Closely related to this, it has been proposed that the impact of naringenin on microbiota composition can also contribute to the beneficial effects exerted by this compound in intestinal inflammation. In this sense, naringenin has been reported to inhibit both growth and adhesion of *Salmonella typhimurium*, a Gram-negative pathogen, to cultured human Caco-2 cells [130]. On the contrary, the same study revealed that this flavanone enhanced the proliferation and adhesion of the probiotic *L. rhamnosus*; of note, this probiotic has been described to exert beneficial effects in human intestinal inflammation [131]. Moreover, it has also been reported that EGCG shows antimicrobial effects and capacity to disrupt bacterial growth, which may also have a positive impact on colonic inflammation [56,132].

## 4. Conclusions

The different studies performed with flavonoids focused on their intestinal anti-inflammatory effect can definitively support their consideration as potential treatments for human IBD. In fact, flavonoids have shown efficacy in experimental models and their mechanisms of action are similar to those described for drugs currently used in human therapy [133]. Besides, their consumption in edible vegetables from ancient times makes them a safe strategy. However, there is a clear demand for clinical trials to confirm their actual role in the treatment of these intestinal inflammatory conditions. Most of the studies carried out in humans consider the use of plants containing flavonoids, and they have shown that these plants may induce clinical remission and clinical response in patients with IBD, although more studies including larger number of patients are required to achieve more solid conclusions [134]. Nevertheless, a pilot study has been carried out to evaluate the efficacy of the oral administration of EGCG and has reported beneficial effects on UC patients refractory to 5-ASA and/or azathioprine [135]. Further clinical studies should definitely be done to elucidate the efficacy and safety of the different flavonoids in these intestinal inflammatory conditions and to finally consider them as solid therapeutic strategies.

## Figures and Tables

**Table 1:** Flavonoids’ intestinal anti-inflammatory effects.

Chemical Class	Plant Source	Mechanism	References
**Anthocyanins**			
Cyanidin-3-glucoside	*Hibiscus sabdariffa* *Hibiscus sabdariffa*	Inhibition PGE_2_ release by regulating COX-2 activity.	[37]
		Reduction pro-inflammatory mediator production.	
		Inhibition STAT pathway.	
**Chalcones**			
Cardamomin	*Alpinia katsumadai Alpinia conchigera*	Inhibition leukocyte migration.	[38]
		Inhibition reactive nitrogen species generation.	
		Reduction pro-inflammatory mediators.	
		Inhibition NF-κB activity.	
**Flavanones**			
Naringenin	Grapefruit (*Citrus paradise*)	Inhibition COX-2 activity.	[39]
		Inhibition leukocyte migration.	[36]
		Inhibition reactive nitrogen species generation.	[40]
		Reduction pro-inflammatory mediator production.	[35]
		Inhibition NF-κB activity.	[41]
		Improvement epithelial barrier function.	[42]
		Antimicrobial effects and gut microbiota modulation.	[43]
**Flavones**			
Chrysin	*Picea crassifolia*	Inhibition leukocyte migration.	
		Inhibition reactive nitrogen species generation.	[39]
		Reduction pro-inflammatory mediators.	[44]
		Inhibition NF-κB activity.	
Baicalin	*Scutellaria baicalensis*	Modulation T cell activity.	[45]
		Inhibition NF-κB activity.	[46]
**Flavonols**			
Quercetin	*Dysosma veitchii*	Inhibition reactive nitrogen species generation.	[47]
		Reduction pro-inflammatory mediator production.	
		Inhibition NF-κB activity.	
Rutin	*Ruta graveolens*	Inhibition COX-2 activity.	
		Inhibition leukocyte migration.	[48]
		Reduction pro-inflammatory mediators.	[49]
		Inhibition NF-κB activity.	
		Improvement epithelial barrier function.	
Quercitrin	Tartary buckwheat (*Fagopyrum tataricum*) Oaks species (*Quercus* sp.)	Inhibition leukocyte migration.	
		Inhibition reactive nitrogen species generation.	[50]
		Reduction pro-inflammatory mediator production.	[34]
		Inhibition NF-κB activity.	
		Improvement epithelial barrier function.	
**Flavanols**			
Epigallocatechin-3-gallate	*Camellia sinensis*	Inhibition COX-2 activity.	
		Inhibition leukocyte migration.	
		Inhibition reactive nitrogen species generation.	[35]
		Reduction pro-inflammatory mediator production.	[41]
		Inhibition NF-κB activity.	[51]
		Inhibition MAPK pathway.	[36]
		Antimicrobial effects and gut microbiota modulation.	[52]
**Isoflavones**			
Daidzein	*Pueraria mirific* *Pueraria lobata* *Glycine max*	Inhibition reactive nitrogen species generation.	[41]
		Inhibition NF-κB activity.	
Glabridin	*Glycyrrhiza glabra*	Inhibition reactive nitrogen species generation.	[53]
Genistein	*Glycine max*	Inhibition leukocyte migration.	[54]
		Reduction pro-inflammatory mediator production.	

## Abbreviations

APCs, antigen-presenting cells; 5-ASA, aminosalicylic acid; BMDM, bone marrow-derived macrophage; CAT, catalase; CD, Crohn’s disease; cNOS, constitutive nitric oxide; COX, cyclooxygenase; C3G, cyanidin-3-glucoside; DC, dendritic cells; DSS, dextran sulphate sodium; EGCG, epigallocatechin-3-gallate; GSH, glutathione; HETE, hydroxyeicosatetraenoic acid; IBD, inflammatory bowel disease; ICAM, intercellular adhesion molecule; IL, interleukin; iNOS, inducible nitric oxide synthase; KC, keratinocyte chemoattractant; LFA, lymphocyte function-associated antigen; LOX, lipoxygenase; LPS, lipopolysaccharide; Mac-1; macrophage 1 antigen; MAPK, mitogen-activated protein kinase; MCP, monocyte chemoattractant protein; MDA, malondialdehyde; MIP, macrophage inflammatory proteins (MIP); MPO, myeloperoxidase; NADPH, nicotinamide adenine dinucleotide phosphate; NF-κB, nuclear factor-κB; NK, natural killer cells; PLA_2_, cytoplasmatic phospholipase A_2_; RNS, reactive nitrogen species; ROS, reactive oxygen species; SOD, superoxide dismutase; Th, T helper; TLR, toll-like Receptor; TNBS, trinitrobenzene sulfonic acid; TNFα, tumour necrosis factor α; UC, ulcerative colitis; VCAM, vascular cell adhesion molecule; VLA, very late antigen.
